# Augmented Input Reveals Word Deafness in a Man with Frontotemporal Dementia

**DOI:** 10.3233/BEN-2012-0356

**Published:** 2012-03-16

**Authors:** Chris Gibbons, Barry Oken, Melanie Fried-Oken

**Affiliations:** ^1^Department of NeurologyOregon Health and Science UniversityPortlandORUSA; ^2^Behavioral NeuroscienceOregon Health and Science UniversityPortlandORUSA; ^3^Biomedical EngineeringOregon Health and Science UniversityPortlandORUSA; ^4^Otolaryngology (ENT), Oregon Health and Science UniversityPortlandORUSA

**Keywords:** Word deafness, augmentative communication

## Abstract

We describe a 57 year old, right handed, English speaking man initially diagnosed with progressive aphasia. Language assessment revealed inconsistent performance in key areas. Expressive language was reduced to a few short, perseverative phrases. Speech was severely apraxic. Primary modes of communication included gesture, pointing, gaze, physical touch and leading. Responses were 100% accurate when he was provided with written words, with random or inaccurate responses for strictly auditory/verbal input. When instructions to subsequent neuropsychological tests were written instead of spoken, performance improved markedly. A comprehensive audiology assessment revealed no hearing impairment. Neuroimaging was unremarkable. Neurobehavioral evaluation utilizing written input led to diagnoses of word deafness and frontotemporal dementia, resulting in very different management. We highlight the need for alternative modes of language input for assessment and treatment of patients with language comprehension symptoms.

## 1. Introduction

Word deafness is characterized by the inability to comprehend and reproduce speech in spite of an apparently functional auditory system. Patients may retain other aspects of language processing such as reading, writing, and spontaneous speech, suggesting that there may be some problem mapping auditory language input to stored lexicon [1–3]. There is disagreement regarding the relevant cognitive and neurophysiologic mechanisms involved. Functionally, the temporal analysis of speech has been implicated by some researchers [4, 5], while others have suggested that the phonetic analysis of speech is affected [6,7]. Bilateral lesions of the temporal lobe [8–10] as well as unilateral lesions of the left posterior temporal lobe [11,12] have been cited. This discrepancy in presumed underlying mechanisms may suggest different types of word deafness, degrees of impairment, or word deafness as a symptom complex rather than pure word deafness in isolation of other related impairments [13–15]. Functionally, word deafness can potentially confound clinical assessment and rehabilitation by limiting accurate communication [16, 17]. Non-auditory language input (i.e., written words, pictures or symbols) offers an alternative route with which to bypass impaired auditory comprehension in cases where visual understanding of language remains intact.

The use of augmentative and alternative communication (AAC) is well established as an intervention strategy for patients with speech and language impairments resulting from amyotrophic lateral sclerosis, traumatic

ISSN 0953-4180/12/$27.50 © 2012 – IOS Press and the authors. All rights reserved

Table 1
Initial assessment raw scores

| Assessment | Score |
|---|---|
| Oral/Verbal agility (subtest of Boston Diagnostic Aphasia Examination) | 0/26 |
| Boston Naming Test | 40/60 |
| Functional reading (subtest of Reading Comprehension Battery for Aphasia) | 6/10 |
| Matrix reasoning (subtest of Weschler Adult Intelligence Scale – IV) | 8/26 |
| The Pyramids and Palm Trees Test | 37/52 |
| Trail Making Test Part B | 100% |
| Western Aphasia Battery Subtests: | |
| Auditory word recognition | 27/60 |
| Sequential commands | 21/80 |
| Repetition | 16/100 |
| Object naming | 56/60 |
| Word fluency | 1/20 |
| Sentence completion | 0/10 |
| Responsive speech | 0/10 |

brain injury, chronic aphasia, and primary progressive aphasia [18–20]. AAC approaches range from picture or photo arrays to adapted computers with a variety of complex language and access technologies. Although AAC is primarily characterized as a means for language expression, it can be considered as a language input technique [21]. Augmented input refers to a technique whereby a conversational partner assists with comprehension by providing written or picture support to the language impaired individual [22,23]. This technique is particularly useful for patients with suspected deficits in auditory comprehension or language processing [24].

## 2. Case report

A 57 year old right handed, English speaking man, with a bachelor's degree in music, had his first neurological evaluation for a degenerative disorder at age 53. His symptoms began 6 months earlier and consisted of difficulty understanding speech, word finding problems, and behavioral changes. His wife reported that he would go up to strangers and shake their hands, and often struggled with language comprehension. He exhibited problems managing his piano repair business, and lost interest in piano playing and music. On testing, he was fully oriented and scored a 26/30 on the Mini-Mental State Examination. He had significant language deficits in confrontation naming and verbal fluency, repetition, and comprehension. MRI did not show any atrophy. A diagnosis of possible frontotemporal dementia with behavioral symptoms and primary progressive aphasia (PPA) was made by his treating neurologist.

Two years later, still carrying a diagnosis of PPA, he was referred to our study on AAC intervention for PPA. At that time, he completed a battery of language and cognitive assessments, and results did not fit into a classic symptom complex. Table 1 displays test scores. Confrontation naming tasks resulted in relatively good performance, whereas word recognition and comprehension tasks resulted in exceptionally poor performance. Low scores were obtained on oral agility and repetition, which was consistent with the apraxia of speech often observed in individuals with progressive aphasia [25]. Intelligible expressive language was characterized by a few short, perseverative phrases most frequently displayed during stereotyped and well rehearsed social interaction, such as greetings, "Hello, I'm fine." and expressions of spousal affection, "I love you." All other spontaneous speech was severely apraxic resulting in monotone and mumbled delivery. Spontaneous connected speech was 90% unintelligible. His primary modes of communication included gesture, pointing, gaze, physical touch and leading.

As part of the study protocol, a picture-based communication board was designed for him. The board included 16 picture + word items about sports, a subject of continuing interest, mounted on 11 × 17 inch laminated cardboard. He was taught how to incorporate the customized communication board into a structured conversation. He participated in a trial conversation using a question and answer format designed to elicit pointing or speech. He failed to answer ten questions correctly, though he remained very engaged in conversation. Examination of his pointing and writing revealed that he was linking pictures from the same semantic network. For instance, he matched pictures of professional baseball players with a picture of their stadium via pointing, and then listed players under stadium headings using pencil and paper, suggesting relatively intact semantic processing. This was significant since his performance on a formal test of semantic linking was inconclusive (Pyramids and Palm Trees, 37/52). In retrospect, it is possible that task expectations during formal testing were unclear since all directions were provided verbally at that time. Formal assessment of written language was not attempted since it was not included in our PPA study assessment protocol.

At this point a new communication strategy was introduced based on his clear attempts to connect items on the board. His conversant wrote down simple yes/no questions and asked him to respond. He answered 5/5 questions correctly without hesitation. Written instructions were then provided for communication board training. Targeted board use increased immediately to 100% accuracy in structured conversation.

He was referred for a full audiologic examination, including tympanic membrane mobility, behavioral response to pure tones, otoacoustic emissions, voice recognition, word recognition, and measures of auditory brainstem response. This assessment revealed an intact auditory system with moderate hearing loss noted at high frequencies and severe impairment for word recognition. He pointed correctly to only 1/12 pictures following a voice prompt at 85 dB. This suggests that his inability to respond appropriately to repetition tasks during the formal cognitive and language testing was compromised by word deafness, and not solely due to aphasia and speech apraxia as expected initially. However, word deafness and apraxia do not explain his performance on the functional reading subtest of the Reading Comprehension Battery for Aphasia (6/10).

Since it became clear that the subject did not meet diagnostic criteria for one of the typical PPA syndromes [26,27], he was excused from participation in our study. In light of his accurate responses using augmented input, he was referred to a behavioral neurologist for evaluation. The consultation was conducted using written input in an effort to more accurately characterize his clinical presentation. Written input produced markedly different testing behavior when compared to spoken input. During his initial assessment, he exhibited very poor performance on tests of oral/verbal agility, sentence completion, auditory word recognition, word fluency, verbal comprehension, and naming tasks, suggesting moderate to severe deficits in these areas. Results on the Neurobehavioral Cognitive Status Exam following written instruction and allowing multiple choice responses, revealed full orientation, intact comprehension of 2-step commands, normal visual naming, near perfect performance on the visual memory task, correct completion of 2 of the 3 visual construction tile designs, and relatively intact mental arithmetic (correct up to 39 divided by 3). Further testing revealed a marked impairment in comprehension of speech, with a concomitant impairment of spontaneous speech, consistent use of yes/no (best when pointing to words on a board), and only mildly abnormal performance on a clock-drawing test. Handwriting was impaired. Overall, results of the neurologic assessment suggest a dementia syndrome with a very marked language disturbance for auditory comprehension as well as expression, likely resulting from frontotemporal degeneration. Based on his conversational behavior with AAC visual supports, as well as the audiologic, neurologic and formal speech-language pathology assessments discussed above, it was determined that he presents clinically with frontotemporal dementia syndrome with aphasia and word deafness.

## 3. Discussion

The use of alternative formats for language comprehension to assist with differential diagnosis and management is highlighted in this case of augmented input to a patient exhibiting irregular comprehension. Results of prior neurological evaluations, language and cognitive tests, as well as observations of functional communication output using an AAC system were equivocal. When examiners used standard spoken instructions, he appeared to present with a severe cognitive dysfunction because his performance on some tests was compromised by his inability to comprehend auditory directions. The introduction of written messages supported full orientation, task comprehension, conversational turn-taking and participation in neurobehavioral assessment. When the neurologist presented all stimuli in the written mode as augmented input with AAC visual supports, he could complete the evaluation successfully. In this case, the use of written instruction during audiologic and neurologic evaluation proved to be critical for accurate diagnosis and highlighted areas of intact cognition. Clinically, written input strategies have been successfully implemented with key communication partners at home and in his supported living environment to enhance comprehension and participation. His spouse and care staff have been taught to write things down as AAC input during social communication, listing daily activities, choosing sporting events to watch on television, meals, and medical decision-making. This case illustrates the effectiveness of augmented input during assessment as well as future medical management and family communication when a patient presents with inconsistent language comprehension.

We acknowledge the support of grants # P30AG0080 17 (National Institute of Health), #H133G080162 and #H133E080011 (National Institute for Disability and Rehabilitation Research, U.S. Department of Education) for funding the research and study of this patient.

## References

[1] T.C. Hain, Cranial nerve 8: Vestibulocochlear system, in: *Textbook of Clinical Neurology*, (3nd ed.), C.G. Goetz, ed., Philadelphia: W.B. Saunders Co.; 2003, pp. 199–216.

[2] K. Hayashi and R. Hayashi, Pure word deafness due to left subcortical lesion: Neurophysiological studies of two patients, *Clin Neurophys* **118** (2007), 863–868.

[3] J.A. Riley and G.B. Cogan, A two mechanism model of pure word deafness, in: *University for Maryland Working Papers in Linguistics*, A. Omaki, J. Ortega-Santos and M. Wagers, eds, College Park, MD: UMWPiL, 2007, pp. 201–221.

[4] M. Albert and D. Bear, Time to understand. A case study of word deafness with reference to the role of time in auditory comprehension, *Brain* **97** (1974), 373–384.

[5] D.P. Phillips and M.E. Farmer, Acquired word deafness, and the temporal grain of sound representation in the primary auditory cortex, *Behav Brain Res* **40** (1990), 85–94.

[6] A. Caramazza, R.S. Berndt and A.G. Basili, The selective impairment of phonological processing: A case study, *Brain and Language* **18** (1983), 128–174.

[7] P. Praamstra, P. Hagoort, B. Maassen and T. Crul, Word deafness and auditory cortical function, *Brain* **114** (1991), 1197–1225.

[8] S.H. Auerbach, T. Allard, M. Naeser, M.P. Alexander and M.L. Albert, Pure word deafness. Analysis of a case with bilateral lesions and a defect at the prephonemic level, *Brain* **105** (1982), 271–300.

[9] R.M. Bauer and T. Zawacki, Auditory agnosia and amusia, in: *Patient-Based Approaches to Cognitive Neuroscience Issues in Clinical and Cognitive Neuropsychology*, M.F. Farah and T.E. Feindberg, eds, Cambridge, MA: the MIT Press; 2000, pp. 97–106.

[10] D. Poeppel, Pure word deafness and the bilateral processing of the speech code, *Cognitive Science* **25** (2001), 679–691.

[11] N. Takahashi, M. Kawamura, H. Shinotou, K. Hirayama, K. Kaga and M. Shindo, Pure word deafness due to left hemisphere damage, *Cortex* **28** (1992), 295–303.

[12] E. Wang, R.K. Peach, Y. Xu, M. Schneck and C. Manry, Perception of dynamic acoustic patterns by and individual with unilateral verbal auditory agnosia, *Brain and Language* **73** (2000), 442–455.

[13] M. Otsuki, Y. Soma, M. Sato, A. Homma and S. Tsuji, Slowly progressive pure word deafness, *Euro Neuro* **39** (1998), 135–140.

[14] L.R. Slevc, C. Martin, A.C. Hamilton and M.F. Joanisse, Speech perception, rapid temporal processing, and the left hemisphere: A case study of unilateral pure word deafness, *Neruopsychologia* **49** (2011), 216–230.

[15] G.A. Stefanatos, A. Gershkoff and S. Madigan, On pure word deafness, temporal processing, and the left hemisphere, *Journal of the International Neuropsychological Society* **11** (2005), 456–470.

[16] I. Rapin, Cortical deafness, auditory agnosia, and word-deafness: How distinct are they? *Human Communication Canada* **9** (1985), 29–37.

[17] G.M. Seliger, F. Lefever, R. Lukas, J. Chen, S. Schwartz, L. Codeghini and G. Abrams, Word deafness in head injury: Implications for coma assessment and rehabilitation, *Brain Injury* **5** (1991), 53–56.

[18] D. Beukelman, S. Fager, L. Ball and A. Deitz, AAC for adults with acquired neurological conditions: A review, *Aug and Alt Comm* **23** (2007), 230–242.

[19] L. Ball, D.R. Beukelman and G. Pattee Communication effectiveness of individuals with amyotrophic lateral sclerosis, *J Comm Dis* **37** (2004), 197–215.

[20] M. Fried-Oken, C. Rowland and C. Gibbons, Providing augmentative and alternative communication treatment to persons with nonfluent progressive aphasia, *Perspectives on Neurophysiology and Neurogenic Speech and Language Disorders* **20** (2010), 21–25.

[21] L.A. Wood, J. Lasker, E. Siegel-Causey, D.R. Beukelman and L. Ball, Input framework for augmentative and alternative communication, *Aug and Alt Comm* **14** (1998), 261–267.

[22] K.L. Garrett and J.P. Lasker, Adults with severe aphasia, in: *Augmentative and Alternative Communication*, (3rd ed.), D.R. Beukelman and P. Mirenda, eds, Baltimore: Paul H. Brookes Publishing Co; 2005, pp. 467–504.

[23] P. Mirenda, Autism, augmentative communication, and assistive technology: What do we really know? *Focus on Autism and Other Dev Dis* **14** (2001), 141–151.

[24] K.L. Garrett and D.R. Beukelman, Changes in the interaction pattern of an individual with severe aphasia given three types of partner support, *Clin Aphas* **23** (1995), 237–251.

[25] J. Hodges, M. Martinos, S. Woollams, K. Patterson and A. Adlan, Repeat and point: Differentiating semantic dementia from progressive non-fluent aphasia, *Cortex* **44** (2008), 1265–1270.

[26] M.F. Bonner, S. Ash and M. Grossman, The new classification of primary progressive aphasia into semantic, logopenic, or nonfluent/agrammatic variants, *Current Neurology and Neuroscience Reports* **10** (2010), 484–490.

[27] M.M. Mesulam, Primary progressive aphasia, *Alzheimer's Disease and Associated Disorders* **21** (2007), S8–S11.
